# Active-Duty Physicians' Perceptions and Satisfaction with Humanitarian Assistance and Disaster Relief Missions: Implications for the Field

**DOI:** 10.1371/journal.pone.0057814

**Published:** 2013-03-26

**Authors:** Geoffrey J. Oravec, Anthony R. Artino, Patrick W. Hickey

**Affiliations:** 1 The Center for the Study of Traumatic Stress, Uniformed Services University, Bethesda, Maryland, United States of America; 2 Department of Preventive Medicine and Biometrics, Uniformed Services University, Bethesda, Maryland, United States of America; Indiana University and Moi University, United States of America

## Abstract

**Background:**

The United States Department of Defense participates in more than 500 missions every year, including humanitarian assistance and disaster relief, as part of medical stability operations. This study assessed perceptions of active-duty physicians regarding these activities and related these findings to the retention and overall satisfaction of healthcare professionals.

**Methods and Findings:**

An Internet-based survey was developed and validated. Of the 667 physicians who responded to the survey, 47% had participated in at least one mission. On a 7-point, Likert-type response scale, physicians reported favorable overall satisfaction with their participation in these missions (mean  = 5.74). Perceived benefit was greatest for the United States (mean  = 5.56) and self (mean  = 5.39) compared to the target population (mean  = 4.82). These perceptions were related to participants' intentions to extend their military medical service (total model *R* ^2^  = .37), with the strongest predictors being perceived benefit to self (β = .21, *p*<.01), the U.S. (β = .19, *p*<.01), and satisfaction (β = .18, *p*<.05). In addition, Air Force physicians reported higher levels of satisfaction (mean  = 6.10) than either Army (mean  = 5.27, Cohen's *d* = 0.75, *p*<.001) or Navy (mean  = 5.60, Cohen's *d*  = 0.46, *p*<.01) physicians.

**Conclusions:**

Military physicians are largely satisfied with humanitarian missions, reporting the greatest benefit of such activities for themselves and the United States. Elucidation of factors that may increase the perceived benefit to the target populations is warranted. Satisfaction and perceived benefits of humanitarian missions were positively correlated with intentions to extend time in service. These findings could inform the larger humanitarian community as well as military medical practices for both recruiting and retaining medical professionals.

## Introduction

An estimated 210,800 full-time aid workers in humanitarian assistance and disaster response (HADR) serve with the United Nations (UN), the Red Cross/Red Crescent and international non-governmental organizations (NGOs) [1]. When volunteers, national NGOs, government organizations and militaries are included the number of individuals participating in HADR soars into the millions. Low retention rates of those working in the humanitarian sector are a recognized barrier to the delivery of high-quality services [2]–[4]. These reports aggregate aid workers regardless of professional background and may not identify profession-specific differences. Prior studies have attempted to address this issue by asking small groups of health professionals about their perceptions, motivations and concerns regarding humanitarian assignments. These studies suggest that factors involving personal growth and satisfaction, as well as a desire to help others and the community, drive health professionals toward HADR, whereas frustration with the nature of the work, unexpected responsibilities, lack of appreciation or concerns of competence may discourage workers from continuing [2], [5]. To date, however, there has been no large-scale study of healthcare workers engaged in HADR activities to help inform the larger humanitarian community.

The U.S. Department of Defense (DoD) conducts more than 500 missions every year in the context of medical stability operations and disaster relief (MSO-DR) through funding made available by the Defense Security Cooperation Agency as part of Overseas Humanitarian, Disaster and Civic Aid (OHDACA) [6]–[8]. A potentially larger number of missions are planned at the deployed military unit level and occur in combat zones such as Afghanistan and Iraq, but these are funded through other sources and their numbers are not well defined. MSO-DR, defined as military medical missions to reestablish a safe and secure environment, provide essential governmental services, emergency infrastructure reconstruction, and humanitarian relief, encompass a broad range of capacity building, infrastructure development, and direct clinical care activities that have become a central pillar of the DoD's mission in support of U.S. government foreign policy [9], [10]. Active-duty physicians in the Army, Navy and Air Force play a key role in these endeavors, and some studies suggest that nearly half of all uniformed healthcare providers have had some type of MSO-DR experience during their career [11]. The DoD has recently increased its attention to measuring the impact of MSO-DR activities and has sought to align military standards of monitoring and evaluation with those of the international aid community [12]–[16]. In addition, the military is continuously striving to retain qualified physicians to enhance mission effectiveness and promote force health protection; positive experiences on MSO-DR missions may improve this capability [11], [17].

The aims of this study were to identify the specific elements of MSO-DR missions that active-duty physicians perceive to be beneficial, how these perceived benefits relate to overall satisfaction with the missions, and what factors have the strongest associations with retention of qualified medical personnel in the military.

## Methods

### Survey Design

To evaluate active-duty physicians' perceptions of humanitarian missions, a survey instrument was created to assess respondents' beliefs about their most recent humanitarian mission. The survey content addressed four conceptual constructs which the authors believed, based on the extant literature, could be potential factors in influencing individuals' perceptions of humanitarian missions and attitudes towards continued military service. The first construct, “satisfaction,” assessed the extent to which the mission was a positive experience in general, and the degree to which the physician would recommend or participate again if given a choice. Second, “perceived benefit to the United States,” focused on the extent to which the mission was viewed as meeting the strategic objectives of the U.S. and the military. Third, “perceived benefit to the target population,” addressed the extent to which the mission was viewed as meeting the needs of the target population, leaving the population better-off, and increasing collaboration. Lastly, “perceived benefit to self,” assessed the extent to which the mission was viewed as helping one's chance of promotion, professional relationships, personal relationships, or professional skills.

These constructs represent the foundation for evaluating perceived benefits and satisfaction, and each was operationalized through a series of questions related to the given construct. Answer choices for each question ranged from “greatly hurt” to “greatly helped” for the constructs dealing with perceived benefits, and “strongly disagree” to “strongly agree” for satisfaction. All answers were marked on a 7-point, Likert-type response scale, with greatly hurt/strongly disagree assigned a numerical value of 1 and greatly helped/strongly agree assigned a numerical value of 7.

The final survey instrument included 51 items. In addition to the questions assessing the four main constructs and a question measuring intent towards retention on active duty beyond the current service obligation, demographic questions were included. These items addressed respondents' sex, age, marital status, branch of service, medical specialty, rank, and years of service.

### Initial Survey Validation

Following initial item development, the survey instrument was evaluated and revised through subject-matter expert validation, cognitive interviewing, and small-scale pilot testing with members of the Uniformed Services University community who had participated in humanitarian missions. After each stage of the process, items were revised based on the feedback received. As a result of the pilot testing, additional items were written for each construct as a means of improving the construct coverage and internal consistency reliability of the survey instrument.

### Survey Implementation

Each year more than 10,000 physicians serve on active duty in the U.S. military, although this number has steadily decreased over the past decade [18]. For the present study, all active-duty physicians were targeted as there currently exists no convenient or reliable means to identify only those physicians who have participated in humanitarian activities. To recruit physicians for the study, Medical Corps Specialty Consultants (Army, Air Force) or Specialty Leaders (Navy) were contacted requesting their support. Consultants who agreed to participate were asked to forward an electronic link to the survey to all physicians within their specialty. The e-mail sent to participants contained a description of the survey and a statement describing the voluntary nature of the study; the e-mail also included a link to the survey itself. One week after the survey was distributed, the participating Consultants were asked to forward a reminder e-mail to the same group of physicians. The Medical Corps websites for each branch of service list 158 Specialty Consultant and Specialty Leader positions within the DoD, although more than one position is occasionally filled by the same person and others are administrative positions not responsible for a specific medical specialty or subspecialty [19]–[21]. E-mail requests for participation were sent to 130 Consultants and Leaders, and of the 60 who replied, a total of 55 agreed to participate. Because the survey link was forwarded by Consultants and Specialty Leaders on a voluntary basis, the specific number of physicians receiving the survey and the number of medical specialties included is not known.

The study was implemented as a voluntary, anonymous, Internet-based survey that required approximately 10 minutes to complete. Data collection for the survey was performed electronically utilizing a password protected, proprietary survey account (surveymonkey.com). Study participants consisted of all active-duty physicians with Internet access who agreed to take the survey after being forwarded the link by their participating specialty Consultant. Medical students, retired or separated military members, and civilian physicians were excluded from the study. If study participants had not participated in a humanitarian mission in the past, they received an abbreviated version of the questionnaire which included questions on demographics, accession and desire to participate in future missions.

### Ethics Statement

The research protocol was approved by the Institutional Review Board of the Uniformed Services University. All survey data were anonymous and no personally identifiable information was collected. Informed consent was obtained on the first page of the survey which highlighted the purpose of the study, risks and benefits, alternatives to participation, the right to withdraw, and where to obtain more information. Participants gave consent by selecting “yes” which brought them to the electronic survey.

### Statistical Analyses

Prior to analysis, data were screened for accuracy and missing values, and each survey item response pattern was checked for normality. Next, an exploratory factor analysis (EFA) was conducted to examine the factorial validity of the survey. Subscales identified in the EFA were analyzed for internal consistency reliability and a mean score for the items associated with a particular subscale was computed (i.e., the variables were un-weighted composite scores). Descriptive statistics were calculated for all variables and a correlation analysis to explore the bivariate associations among the survey variables was conducted. Multiple linear regression analysis was used to evaluate how well a linear combination of the survey variables could explain the variation in participants' intentions to extend their military service beyond their current commitment. Finally, one-way multivariate analysis of variance (MANOVA) was used to explore whether service membership (i.e., U.S. Air Force, Army, or Navy) was related to participants' scores on the survey variables. All analyses were completed using SPSS 20.0 (IBM Corporation, New York, NY).

## Results

The majority of respondents were from the Air Force, male, 31–45 years old, married, and had more than 10 years of service in the military. Almost half of all respondents had participated in a MSO-DR operation, and of those who had, most had participated in more than one mission. The characteristics of the 667 participants are presented in Table 1.

**Table 1:** Study sample statistics.

Characteristic	Total N (%) or Mean (SD)	Air Force N (%) or Mean (SD)	Army N (%) or Mean (SD)	Navy N (%) or Mean (SD)
Total Respondents	667 (100)*	271 (41)	206 (31)	156 (23)
Participated in MSO-DR Mission	316 (47)**	137 (51)	76 (37)	85 (55)
Male	446 (67)	172 (64)	159 (77)	113 (72)
Age 31–45	430 (65)	206 (76)	122 (59)	101 (65)
Married	535 (80)	231 (85)	170 (83)	131 (84)
Military Rank O4 – O5	394 (59)	176 (65)	118 (57)	99 (64)
Years of Service	13.19 (7.50)	12.92 (6.38)	17.83 (6.54)	15.48 (7.23)
Past Number of Missions	2.37 (2.98)	2.25 (2.59)	2.90 (3.73)	1.94 (1.64)

* The total number of respondents (N = 667) does not equal the sum of the military services because several respondents (N = 32; 5%) failed to indicate their respective service and 2 respondents categorized themselves as Public Health Service. **The total of those who participated in MSO-DR missions (N = 316) does not equal the sum of the military service members who participated in MSO-DR missions because several respondents (N = 18) failed to indicate their respective service.

### Exploratory Factor Analysis

A principal axis factor (PAF) analysis with oblique rotation (Oblimin; delta  = 0) was conducted on the 26 survey items that made up our four constructs of interest (see factor analysis recommendations in Preacher & MacCallum, 2003) [22]. Oblique rotation methods allow for factors to be correlated, and we assumed the four hypothesized factors were related. Evaluation of the correlation matrix indicated that it was factorable: Kaiser-Meyer-Olkin Measure of Sampling Adequacy was .94, which is “marvelous” (>.90) according to Kasier's criteria [23]. Bartlett's Test of Sphericity (*χ* ^2^  = 6700.72, *df*  = 325, *p*<.001) was significant, indicating that the correlation matrix was not an identity matrix, and all measures of sampling adequacy were deemed sufficient (i.e., >.60) [23].

The number of factors to extract was determined using several criteria, including parallel analysis, examination of the resulting scree plot, and eigenvalues greater than 1.0 [24]. All three criteria suggested a five-factor solution, with the five factors accounting for 67% of the total variance in the items. Inspection of the table of communalities revealed that all but one item had high extracted communalities (i.e., >.40), which indicates that much of the common variance in the items can be explained by the five extracted factors [23]. The one exception, BS-1, had a low extracted communality (.16).

Several additional rules were used to determine the number of factors and individual items to retain in the final solution: (a) factors needed to contain at least three items; (b) the absolute value of all factor pattern coefficients needed to be >.35 on at least one factor; and (c) items with factor pattern coefficients (absolute value) ≥.30 on more than one factor were dropped (see recommendations in Pett et al., 2003) [23].

The factor pattern coefficients from the PAF analysis of the four survey constructs are displayed in Table 2, including the specific questions associated with each factor. The first factor addressed general feelings of satisfaction (SAT) (extraction eigenvalue  = 12.52) and included six items: SAT-1, SAT-2, SAT-3, SAT-4, SAT-5, and SAT-6. The second factor, benefit of the mission to the United States (BU) (extraction eigenvalue  = 1.96) included five items: BU-1, BU-2, BU-3, BU-4, and BU-5, and the third factor, benefit to the target population (BT) (extraction eigenvalue  = 1.81) also included five items: BT-2, BT-3, BT-4, BT-5, and BT-6. Although item BT-1 loaded moderately on Factor 3, it also loaded highly on Factor 1 (and had a low extracted communality); it was therefore dropped from the final solution. Factors four and five were derived from our fourth survey construct, benefit to self (BS). The fourth factor (extraction eigenvalue  = 1.68) included three items: BS-2, BS-3, and BS-4, and the fifth factor (extraction eigenvalue  = 1.08) included four items: BS-5, BS-6, BS-8, and BS-9. Although item BS-7 loaded moderately on Factor 5, it also loaded on Factors 1 and 2; it was therefore dropped from the final solution.

**Table 2:** Results (pattern coefficients) from the EFA with Oblique Rotation (Oblimin; delta  = 0) on the 26 survey items (*N* = 308).

Item	Factor				
	1	2	3	4	5
SAT-1 I enjoyed participating in this humanitarian mission.	**.69**	.12	−.04	.01	−.22
SAT-2 Overall, I was satisfied with this humanitarian mission.	**.80**	.06	.02	.02	-.10
SAT-3 I was satisfied with the type of work I conducted on this mission.	**.88**	−.01	.08	.05	.03
SAT-4 I was satisfied with the amount of work I did on this mission.	**.84**	−.02	.05	.02	.01
SAT-5 I was satisfied with the interactions I had with the target population.	**.70**	.16	.05	−.01	−.03
SAT-6 I would recommend participating in a humanitarian mission, such as my most recent mission, to a friend.	**.77**	.10	−.04	−.01	−.18
BU-1 The image of the United States as a country?	.05	**.91**	.06	−.01	.06
BU-2 The image of the U.S. military?	.01	**.95**	.02	−.01	.08
BU-3 The image of American physicians?	.15	**.74**	−.02	.13	−.03
BU-4 The image of American military medicine?	.08	**.90**	−.03	−.01	.04
BU-5 The likelihood of future cooperation between the governments of the host nation and the U.S.?	−.07	**.80**	.05	.01	−.10
BT-1 The majority of target population individuals that were seen?	.47	.07	.34	.11	.19
BT-2 The medical capability (knowledge, skills) of target population health care workers?	−.05	.01	**.77**	.01	−.12
BT-3 The medical practice (demand for services, livelihood) of the target population health care workers?	−.02	−.02	**.90**	−.02	−.05
BT-4 The target population health care system?	.12	−.01	**.79**	.02	.09
BT-5 The target population as a whole?	.28	.08	**.61**	.06	.21
BT-6 Collaboration between U.S. healthcare workers and target population healthcare workers?	−.04	.22	**.62**	−.02	−.20
BS-1 Your chance of promotion?	−.09	.16	.12	.25	−.06
BS-2 Your relationship with your family?	.05	−.08	−.02	**.70**	.01
BS-3 Your professional relationships with co-workers at your home station?	−.03	.11	−.04	**.62**	−.03
BS-4 Your relationships with your friends outside of work?	−.01	−.02	−.01	**.80**	.04
BS-5 Your professional skills (those skills that you use in your daily job at your home station)?	.19	−.06	.09	.29	−**.36**
BS-6 Your overall sense of well-being (how you feel about yourself as a person)?	.26	.16	.11	.21	−**.38**
BS-7 Your sense of professional pride in being a military physician?	.31	.37	−.04	.05	−.35
BS-8 Your desire to interact with foreign cultures in the future?	.22	.01	.04	.03	−**.66**
BS-9 Your confidence in being able to practice medicine in environments unlike those found in the United States?	.12	.04	.25	.06	−**.49**

*Note*. Entries in bold indicate pattern coefficients (absolute values) >.35 on at least one factor and pattern coefficients (absolute values) ≥.30 on only one factor.

### Factor Labels, Reliability Analysis, and Subscale Creation

Based on EFA results, five factors remained in the final solution: (a) Factor 1 was labeled *satisfaction*, (b) Factor 2 was labeled *benefit to the U.S.*, (c) Factor 3 was labeled *benefit to the target population*, (d) Factor 4 was labeled *benefit to relationships*, and (e) Factor 5 was labeled *benefit to self*.

Cronbach's alpha coefficients for each of the five subscales were used to assess the internal consistency reliability of the scores. As indicated in Table 3, all alpha coefficients were well within the desired range, with actual values of .74−.95 (see guidelines in Gable & Wolfe, 1993) [25]. Composite variables were used in subsequent analyses. These variables were created by computing a mean score for the items associated with a particular subscale.

**Table 3:** Descriptive statistics, Cronbach's alphas, and Pearson correlations between the five survey variables and participants' intentions to extend their military service beyond their current commitment.

Variables	Means	*SD*	No. of Items	Cronbach's Alpha	1	2	3	4	5	6
1. Satisfaction	5.74	1.25	6	.95	–	.62	.61	.35	.70	.53
2. Benefit to U.S.	5.56	.99	5	.96		–	.59	.38	.53	.50
3. Benefit to target population	4.82	.87	5	.90			–	.32	.54	.46
4. Benefit to relationships	4.24	.68	3	.74				–	.43	.34
5. Benefit to self	5.39	.93	4	.83					–	.53
6. Intentions to extend military service	4.12	1.65	1	–						–

*Note*. All survey variables were measured using a 7-point, Likert-types response scale, and all correlations are significant at the *p*<.001 level.

### Descriptive Statistics and Correlation Analysis

Table 3 presents the means and standard deviations of the HADR subscale variables and the individual item used as an outcome in the multiple regression (“Because of this most recent humanitarian mission, I am more likely to extend my service in the military beyond my current commitment”). Correlations between these variables are also presented. As shown, all of the correlations were statistically significant at the *p*<.001 level. In particular, participants' satisfaction with the mission was positively correlated with their self-reported benefit to the U.S. (*r* = .62), benefit to target population (*r* = .61), benefit to relationships (*r* = .35), benefit to self (*r* = .70), and intentions to extend military service (*r* = .53). Furthermore, self-reported benefit to U.S. was positively correlated with benefit to target population (*r* = .59), benefit to relationships (*r* = .38), benefit to self (*r* = .53), and intentions to extend military service (*r* = .50). Similarly, self-reported benefit to target population was positively correlated with benefit to relationships (*r* = .32), benefit to self (*r* = .54), and intentions to extend military service (*r* = .46). Moreover, self-reported benefit to relationships was positively correlated with benefit to self (*r* = .43) and intentions to extend military service (*r* = .34). Similarly, self-reported benefit to self was positively correlated with intentions to extend military service (*r* = .53).

### Multiple Regression Analysis

Table 4 presents results from the multiple linear regression using the survey variables to explain the variation in participants' intentions to extend their military service beyond their current commitment. To test for excessive multicollinearity, the correlation table was inspected and tolerance and inflation factor values were calculated. Findings indicated that the absolute values of all the Pearson correlations were ≤.70 (greater than .80 may indicate a problem); the tolerance values were all greater than .40 (less than .10 is evidence of a serious problem); and the variance inflation factors were all less than 2.5 (greater than 10 is evidence of a serious problem; see guidelines in Cohen et al., 2003) [26]. Based on these findings, it was determined that each independent variable had the potential to explain unique variance in the dependent variable (intentions to extend military service).

**Table 4:** Model Summary for the Regression Analysis of Participants' Intentions to Extend their Military Service (*N* = 308).

Independent Variable	*B*	*SE B*	β	*p*-value
Satisfaction	.24	.10	.18	.014
Benefit to U.S.	.31	.11	.19	.003
Benefit to target population	.18	.12	.09	.134
Benefit to relationships	.20	.13	.08	.122
Benefit to self	.39	.12	.21	.002

Results from the multiple regression indicated that the model was statistically significant, *F*(5, 297)  = 35.14, *p* <.001, with the five survey variables explaining 37% of the variance in participants' intentions to extend their military service (a large effect size). The strongest individual predictors of intent to extend military service were benefit to self (β = .21, *p*<.01) and benefit to U.S. (β = .19, *p*<.01). Satisfaction with the mission was also a significant individual predictor of participants' intentions to extend their military service (β = .18, *p*<.05). Somewhat surprisingly, benefit to target population was not a significant individual predictor of intent to extend military service (β = .09, *p* = .134).

### Group Comparisons by Service

**Figure 1:**
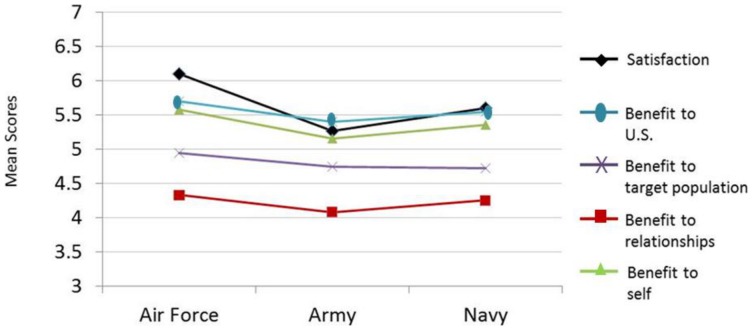
Group comparisons by service. Plots of mean scores for participants' self-reported satisfaction, benefit to U.S., benefit to target population, benefit to relationships, and benefit to self (organized by service). Statistically significant differences were found between the services for satisfaction, benefit to relationships, and benefit to self. All survey variables were measured using a 7-point, Likert-type response scale.

*Note*. *R* ^2^ = .37.

Results from the MANOVA indicated that participants in different services had significantly different scores on several of the survey variables, *F*(10, 582)  = 3.10, *p*<.001. As the overall *F-*test was statistically significant, additional univariate analyses were conducted [27]. Tests of between-subjects effects indicated that branch of service was related to satisfaction (*F*(2, 295)  = 12.48, *p*<.001), benefit to relationships (*F*(2, 295)  = 3.69, *p*<.05), and benefit to self (*F*(2, 295)  = 5.40, *p*<.01). These analyses of variance tests were followed-up with Tukey's HSD post-hoc tests, which indicated that Air Force personnel reported statistically significantly higher levels of satisfaction (Mean  = 6.10) than both their Army (Mean  = 5.27) and Navy (Mean  = 5.60) counterparts. The effects for the differences between the Air Force and Army were moderate (Cohen's *d*  = 0.75, *p*<.001), as were the differences between the Air Force and Navy (Cohen's *d* = 0.46, *p* < .01). Air Force personnel also reported higher scores on benefit to relationships (Mean = 4.34) and benefit to self (Mean  = 5.56), but these differences were only statistically significantly different from Army scores (Mean  = 4.08 and 5.16, respectively). The effects for the differences between the Air Force and Army on benefit to relationships and benefit to self were both moderate (Cohen's *d* = 0.43 and .37, respectively). These group differences are depicted in Figure 1.

## Discussion

Humanitarian assistance missions and military medical stability operations have the potential to reduce suffering, save lives, develop healthcare resources, and stabilize regions. The success or failure of these endeavors, however, depends largely on the healthcare workers who are responsible for carrying them out. Prior studies have attempted to characterize the motivations of humanitarian volunteers or the demands and stresses of the field that might affect mission success. A review of the literature suggests, however, that the present study is the first to examine the satisfaction and perceived benefits of a large population of physicians working in humanitarian assistance and disaster relief [2], [5], [28].

In the current study, a survey instrument with evidence of reliability and validity was developed using a systematic survey development process. The survey was created to evaluate perceived benefits and satisfaction among active-duty physicians participating in HADR activities. Exploratory factor analysis revealed that the vast majority of responses to the questions on perceived benefits of HADR activities could be explained by five underlying factors, derived from the four conceptual constructs identified during survey development: benefit to the United States, benefit to the target population, benefit to relationships, benefit to self, and overall satisfaction with the mission. Each of these five constructs was shown to have good internal consistency reliability.

While this study surveyed a sample of active-duty military physicians, the constructs developed are not military specific. Although military HADR activities may ultimately be related to larger organizational and strategic objectives, the individual physicians carrying out those missions are most likely focused on providing quality services within their field of expertise. In this manner, it is reasonable to consider that both military and non-military humanitarian physicians might experience similar stressors and rewards from these activities. The survey instrument designed in this study could be applied to other international, governmental or non-governmental organizations working in the field as a way of generalizing perceived benefits and satisfaction of humanitarian workers. Such data will be critical as these subjective measures may inform organizational leaders of how their staff perceives the role they play and the organization's contribution, which in turn could directly impact the retention of qualified workers in the humanitarian sector. High employee and volunteer turnover rates among humanitarian workers can impede the continuity of services provided. More studies using similar instruments are needed as the number of studies examining these human factors to date has been small. This information should be shared with other humanitarian actors, as well as the larger scientific community, to help improve ongoing and future humanitarian activities in which satisfaction and retention of workers might also be considered part of a monitoring and evaluation framework.

This study demonstrates that active-duty physicians perceive some benefit to themselves, their relationships, the target population, and the United States from participating in humanitarian activities; but that the largest perceived benefit is to themselves and the U.S. Our assumption was that health professionals involved in humanitarian work would be primarily interested in saving lives and reducing suffering. As such, we expected to see a higher perceived benefit to the target population. Although this finding may be reflective of a sampling bias in that military physicians may have different motivations than other humanitarian workers in the field, it more likely reflects the fact that motivations for participation are not directly linked to perceived impact, which can be affected by a number of other external factors. It may also reflect the type of HADR missions in which the military is involved, and the varying strategic goals associated with these types of activities. At what point the deviation of expectations from perceived impact becomes detrimental to job retention merits further evaluation, as better understanding of this issue could improve recruiting and training efforts.

Overall, active-duty physicians reported feeling satisfied with their most recent humanitarian mission. The two largest perceived benefits from participating in HADR activities, benefit to self and benefit to the U.S., were highly correlated with reported satisfaction. Interestingly, benefit to the target population was also highly correlated with satisfaction. This suggests that even if physicians did not feel that HADR missions were as beneficial to the target population as they were to themselves or the U.S., any perceived benefit to the target population was still strongly related to physicians' views on the most recent mission. Satisfied doctors may be more likely to remain in the humanitarian field, more likely to participate in future missions and less likely to “burnout” from the stress of HADR activities. Factors that increase satisfaction should be considered when planning and developing objectives for humanitarian missions. These factors could impact mission effectiveness and help maintain a qualified workforce.

Satisfaction with the most recent humanitarian mission was shown to be a statistically significant predictor of active-duty physicians' intentions to extend their current military commitment. Physicians who reported perceiving a greater benefit to themselves or to the U.S. also reported greater intentions to extend their time in service. These findings suggest that missions that are more beneficial to the individual physician and that are seen as enhancing the image of the U.S. may positively impact retention. Emphasizing these factors might therefore have a positive effect on maintaining experienced physicians in the military. While in this study the question about retention specifically referred to military service, the same findings may hold true for other humanitarian workers choosing to remain with their organization or participate in future HADR operations (although additional research specifically testing this claim is clearly needed). Surveys of employees departing the International Committee of the Red Cross, from varied professional backgrounds, clearly show personal life motivations and satisfaction as a major factor in the decision [4]. Satisfaction was shown to be correlated with benefits to the physician, the U.S. and the target population. This raises the question of whether retention could be even further improved if missions were designed to maintain a benefit for the individual and the U.S., while at the same time increasing the benefit to the target population. Additional research to identify the attributes of a mission that specifically enhance physician satisfaction needs to be conducted and should be incorporated into organizational measures of effectiveness.

Finally, the survey revealed statistically significant differences in participant scores on satisfaction, benefit to self and benefit to relationships when comparing between the three services. Air Force personnel reported higher satisfaction than either of the other two branches, and also had higher perceived benefit to self and relationships compared to the Army personnel. These findings suggest that the Air Force might have an easier time retaining experienced humanitarian physicians compared to the other services. These results also highlight the differences in perceptions and attitudes that can exist between different organizations carrying out HADR activities. Whether this is due to intrinsic differences in organizational structure, practice patterns within the various branches of the military, or a function of the type and duration of missions being carried out requires further study. This is an important consideration in a field where there is an extremely wide variety of organizations often responding to similar disasters and humanitarian crises.

A significant limitation of this study is that the exact survey response rate is unknown. Although 55 Specialty Leaders and Consultants agreed to participate, there was no process for verifying that these individuals actually forwarded the survey on to their respective specialties. In addition, the Specialty Leaders and Consultants did not provide information as to how many physicians actually received the survey. In this manner, we only know how many physicians actually started the survey and how many completed it. Because of this limitation, we cannot rule out selection and/or response bias, which makes generalizable conclusions difficult to draw. If everybody who received the survey completed it, then we might say that response bias was not a great concern. Without this information, however, we cannot be sure that a large group of individuals chose not to participate and cannot evaluate why this might be. This is a concern as those who have strong feelings, either good or bad, about humanitarian missions might be more inclined to complete the survey when compared to those who are more ambivalent about their experience. Mundell's study of military physician retention in the era of combat operations in Iraq and Afghanistan showed that deployments early in a physician's career (but not later) are negatively correlated with retention [17]. Because the design of the study presented here did not include an assessment of intent to remain on active duty for those who had never participated in a HADR activity, we are not able to draw direct parallels.

Another limitation of this study is that the survey was entirely Internet based. This limited the group of physicians who participated to only those with Internet access. While at first glance this might not seem like a real problem, in this case it may have resulted in failure to collect data from physicians currently deployed on humanitarian missions who have no computer access. Input from such doctors would be beneficial, but the protocol had no method for conducting paper surveys in the field.

Finally, the question of whether these results can be generalized to all physicians in the military remains unclear. As already described, not all medical specialties participated in this study and for those that did the number of respondents was quite variable. The results were heavily weighted towards the medical specialties, with a very large number of family physicians and pediatricians responding. Surgical specialties had a much lower number of responses. This clearly limits the generalizability of our findings, as doctors in different specialties may have very different opinions about humanitarian missions. Future work should attempt to address this limitation.

This study represents a first exploration into how different organizational structures or systems, in this case military branch of service, might impact the perceptions and satisfaction of employees and volunteers. Active-duty physicians appear to be largely satisfied with their involvement in MSO-DR missions. Further elucidation of factors that may increase the perceived and real benefit to the target populations of DoD HADR missions is warranted. Satisfaction with and perceived benefits of humanitarian missions were positively correlated with intentions to extend military service. These findings are not only applicable to the DoD, but could inform the larger humanitarian community and inform practices for both recruiting and retaining medical professionals. The authors welcome other researchers to adapt our instrument for use in other settings and with other aid organizations. Further studies are needed to examine this issue and elaborate on how practices might be standardized across various organizations to not only improve perceived benefits, satisfaction and retention rates, but also to deliver more effective assistance that, ultimately, can have a greater impact on the target population.
